# Delegates from the Medical Society of Delaware

**Published:** 1852-01

**Authors:** B. F. Chatham

**Affiliations:** Secretary


DELEGATES FROM THE MEDICAL SOCIETY OF DELAWARE.

At the annual meeting of the Medical Society of Delaware, held at Cantwell's Bridge, June 10th, 1851, the following named gentlemen were elected delegates from this Society to the American Medical Association, for the year 1852.

*New Castle County.*—Drs. James W. Thompson, Lewis P. Bush James Couper.

*Kent County.*—Drs. Isaac N. Jump, John D. Perkins, G. Saulsbury.

*Sussex County.*—Drs. W. W. Wolfe, George W. Maull, W. W Ricards.

B. F. Chatham, *Secretary.*

